# Supplementation of Sulfide or Acetate and 2-Mercaptoethane Sulfonate Restores Growth of the *Methanosarcina acetivorans* Δ*hdrABC* Deletion Mutant during Methylotrophic Methanogenesis

**DOI:** 10.3390/microorganisms11020327

**Published:** 2023-01-28

**Authors:** Alicia M. Salvi, Niaz Bahar Chowdhury, Rajib Saha, Nicole R. Buan

**Affiliations:** 1 Department of Biochemistry, Redox Biology Center, University of Nebraska-Lincoln, Lincoln, NE 68588-0664, USA; 2 Department of Chemical and Biomolecular Engineering, University of Nebraska-Lincoln, Lincoln, NE 68588-0664, USA

**Keywords:** archaea, *Methanosarcina*, heterodisulfide reductase, methane, methanogenesis

## Abstract

Methanogenic archaea are important organisms in the global carbon cycle that grow by producing methane gas. *Methanosarcina acetivorans* is a methanogenic archaeum that can grow using methylated compounds, carbon monoxide, or acetate and produces renewable methane as a byproduct. However, there is limited knowledge of how combinations of substrates may affect metabolic fluxes in methanogens. Previous studies have shown that heterodisulfide reductase, the terminal oxidase in the electron transport system, is an essential enzyme in all methanogens. Deletion of genes encoding the nonessential methylotrophic heterodisulfide reductase enzyme (HdrABC) results in slower growth rate but increased metabolic efficiency. We hypothesized that increased sulfide, supplementation of mercaptoethanesulfonate (coenzyme M, CoM-SH), or acetate would metabolically alleviate the effect of the Δ*hdrABC* mutation. Increased sulfide improved growth of the mutant as expected; however, supplementation of both CoM-SH and acetate together were necessary to reduce the effect of the Δ*hdrABC* mutation. Supplementation of CoM-SH or acetate alone did not improve growth. These results support our model for the role of HdrABC in methanogenesis and suggest *M.acetivorans* is more efficient at conserving energy when supplemented with acetate. Our study suggests decreased Hdr enzyme activity can be overcome by nutritional supplementation with sulfide or coenzyme M and acetate, which are abundant in anaerobic environments.

## 1. Introduction

*Methanosarcina acetivorans* is an anaerobic methanogenic archaeum found in biomass-rich marine sediment. In this environment, *M. acetivorans* is hypothesized to compete with other anaerobic microbes including heterotrophic degraders and sulfate reducers for nutrients. Growth substrates, which may include methanol, carbon monoxide, methylated amines (mon-, di- and trimethylamine), methylsulfides (methanethiol and dimethylsulfide), or acetate, are oxidized to CO_2_ to generate a transmembrane ion gradient for ATP synthesis, with obligate production of methane. A key enzyme in this process is heterodisulfide reductase, Hdr. Hdr is the terminal oxidase in methanogens, which is responsible for reducing the terminal electron acceptor, the heterodisulfide of coenzyme M and coenzyme B (CoM-S-S-CoB) that is formed in the last step pf methanogenesis. Hdr comes in two varieties in *M. acetivorans*: a cytochrome-containing membrane HdrED which accepts electrons from the membrane electron carrier methanophenazine, and the cytoplasmic HdrABC. In *M. acetivorans*, HdrABC comes in two varieties: HdrA1B1C1 is cotranscribed from a single *hdrA1C1B1* (*hdrABC*, MA3126-MA3128) operon and is expressed during growth on methylotrophic substrates, while HdrA2B2C2 is transcribed from two separate operons, MA2867-MA2868 which encodes HdrA2 and a polyferredoxin, and MA4236–4237 which encodes HdrC2B2.

**Figure 1:**
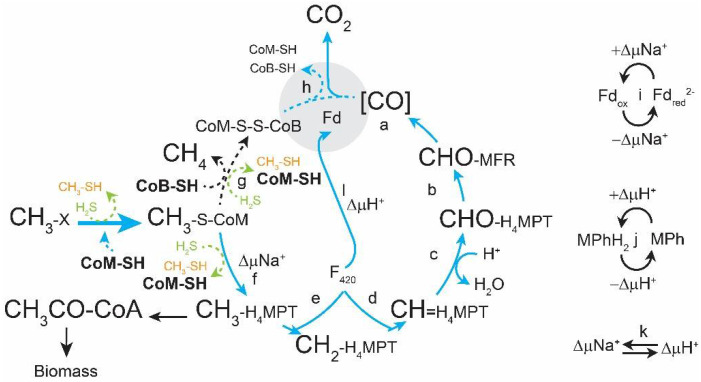
Model for the effect of HdrABC deletion on methylotrophic methanogenesis. When Δ*hdrABC* is deleted, the terminal oxidase reaction that regenerates CoM-SH and CoB-SH cofactors is slowed. As a result, methane formation rate decreases as free CoM-SH and CoB-SH are depleted while CH_3_-CoM and CoM-S-S-CoB accumulates. In addition, CH_3_-Cbl is susceptible to H_2_S in the medium, resulting in formation of CH_3_-SH (orange) and (CH_3_)_2_S, which triggers expression of methylsulfide methyltransferase enzymes. To regenerate CoM-SH, cells up-regulate genes for CoM-SH and CoB-SH biosynthesis (bold) and CoM:methyltransferases (thick blue arrow). The oxidative branch of methylotrophic methanogenesis pathway is shown in blue arrows. CoB-SH, coenzyme B thiol; CoM-SH, Coenzyme M thiol; CoM-S-S-CoB, coenzyme M-coenzyme B heterodisulfide; Fd, ferredoxin; Fd_red_, reduced ferredoxin; H_2_S, hydrogen sulfide (green); H_4_MPT, tetrahydromethanopterin; MFR, methanofuran; MPh, methanophenazine; MPhH_2_, reduced methanophenazine. Enzymes involved in the Wolfe Cycle: (a) formyl-methanofuran dehydrogenase (Fmd), (b) formyl-methanofuran:H_4_MPT formyl transferase (Ftr), (c) methenyl-H_4_MPT cyclohydrolase (Mch), (d) F_420_-dependent methylene-H_4_MPT dehydrogenase (Mtd), (e) F_420_-dependent methylene-H_4_MPT reductase (Mer), (f) methyl-H_4_MPT:coenzyme M methyltransferase (Mtr), (g) methyl-coenzyme M reductase (Mcr), (h) heterodisulfide reductase HdrABC, (i) ferredoxin:methanophenazine oxidoreductase Rnf, (j) Proton-translocating methanophenazine:heterodisulfide reductase (HdrED), (k) Sodium–proton antiporter (MrpA), (l) proton-pumping F_420_H_2_: methanophenazine reductase (Fpo). Figure adapted from [3].

In previous work we observed that when the gene for the methylotrophic-specific HdrA1B1C1 enzyme was deleted (Δ*hdrABC*), cells were still viable and ^13^C and transcriptomic studies suggested the Δ*hdrABC* mutant phenotype was caused by decreased ferredoxin redox cycling and disruption in CoM-SH redox homeostasis (Figure 1) [1]. The Δ*hdrABC* mutant displayed slower rates through the oxidative branch of methylotrophic methanogenesis and increased metabolic efficiency while overexpression of *hdrABC* by integration of a second copy of the *hdrABC* locus caused the opposite phenotype [2]. We hypothesized that reduced flux through ferredoxin in the Δ*hdrABC* mutant also causes methyl-coenzyme M reductase (Mcr) to stall, allowing sulfide in the medium to disrupt Mcr and corrinoid methyltransferases, resulting in production of methanethiol and dimethylsulfide. Accumulation of methanethiol and dimethylsulfide likely induced increased expression of corrinoid methyltransferases in an attempt to (re)capture more substrate. As substrate is converted to methane and sulfide and methanol are depleted, the same corrinoid methyltransferases are used to consume methylsulfides as a carbon and energy source.

In this model, the intracellular concentrations of sulfide, CoM-SH, and reduced ferredoxin are critical parameters that affect cell physiology. We wanted to test whether sulfide, exogenously supplemented CoM-SH, or mixotrophic growth on methanol + acetate would affect the Δ*hdrABC* phenotype as predicted.

## 2. Materials and Methods

### 2.1. Culture Conditions

Strains were obtained from the sources listed in Table 1. Cultures were inoculated anaerobically in a custom B-type Coy anoxic chamber (Coy Labs, Grass Lake, MI, USA) under a 5% H_2/_20% CO_2/_75% N_2_ (±3%) (Matheson Gas, Lincoln, NE, USA) atmosphere and incubated outside of anaerobic chamber are contained in glass Balch tubes secured with butyl rubber stoppers (Bellco Glass, Vineland, NJ, USA) and aluminum crimps (Wheaton, Millville, NJ, USA). Cultures were grown in high salt mineral medium (HS) [200 mM NaCl, 45 mM NaHCO_3_, 13 mM KCl, 54 mM MgCl_2_•6H_2_O, 2 mM CaCl_2_•2H_2_O, 2 µM 0.1% resazurin (w v^−1^), 5 mM KH_2_PO_4_, 19 mM NH_4_Cl, 2.8 mM cysteine•HCl, 0.1 mM Na_2_S•9H_2_O, trace elements, vitamin solution] according to established methods [4] and medium was supplemented with a carbon and energy source (125 mM methanol, or 125 mM methanol plus 40 mM sodium acetate) and 2 mg L^−1^ puromycin as needed at 35 °C. For growth on HS methanol medium, cells were adapted for 30 generations (6 passages of 0.25 mL into 10 mL cultures) before measuring growth rates. Likewise, cells grown on HS methanol + acetate medium, cells were adapted before measuring growth rates. To measure the effect of sulfide concentration, sodium sulfide was varied from 0.25 mM to 0.4 mM in HS medium. For no sulfide medium (0 mM), strains were grown on HS medium without resazurin or sodium sulfide according to established methods [5,6] after adapting for 15 generations (3 passages of 0.25 mL into 10 mL cultures). Culture growth was measured at 600 nm using a Spectronic D spectrophotometer fitted with a Balch tube (18 mm) modification or using a Tecan Sunrise UV/Vis spectrophotometric plate reader.

### 2.2. Strain Validation

After growth curves were completed, strains were checked using a PCR assay as published previously [2]. Primers shown in Table 1 were designed using VectorNTI software (ThermoScientific, Waltham MA, USA). PCR primers were synthesized by Integrated DNA Technologies (IDT, Coralville, IA, USA). The proofreading Phusion Flash PCR Master Mix was used for all PCR amplification (ThermoScientific, Waltham, MA, USA).

### 2.3. Genome-Scale Metabolic Model of Methanosarcina acetivorans

To verify the experimental findings and acquire better understanding of the metabolic trade-offs of the Δ*hdrABC* mutant, we used the most recent genome-scale metabolic model (GEM) of *M. acetivorans*, iST807 [8]. We used parsimonious flux balance analysis (pFBA) to simulate the GEM [9]. pFBA is constrained-based optimization technique to model GEMs. The pseudo-steady state mass balance in pFBA is represented by a stoichiometric matrix, where the columns represent metabolites, and the rows represent reactions. For each reaction, upper and lower bounds are imposed based on Gibbs free energy information. pFBA provided the flux value for each reaction in the model by solving the following optimization problem:

(1) $$\begin{array}{l} \min\;\sum_{j\in J}|v_{j}|\;\\ Subject\;to:\\ \sum_{j\in J}S_{ij}v_{j}=0,\;\;\;\forall i\in \;I\;\;\;\;\;\;\;\;\;\;\;\;\;\;\;\;\;\;\;\; \end{array}$$

(2) $$v_{biomass}=v_{biomass,\;max}\;\;\;\;\;\;\;\;\;\;\;\;\;\;\;\;\;\;$$

(3) $$a_{j}\leq v_{j}\leq b_{j}$$

In this formulation, I is the set of metabolites and J is the set of reactions in the model. $S_{ij}$ is the stoichiometric matrix with i indicating metabolites and j indicating reactions, and $v_{j}$ is the flux value of each reaction. The objective function represents the minimization of sum of absolute values of all fluxes to achieve maximum biomass growth rate, $v_{biomass,\;max}$. From a biological perspective, this objective function along with Equation (3) indicate the most efficient usage of enzyme to reach a certain cellular phenotype which maximizes the biomass production. $LB_{j}$ and $UB_{j}$ are the lower and upper bounds of flux values for each reaction.

In iST807, there are alternate pathways in the electron transport chain. One such reaction is the MTR_BYPASS, which bypassed the actual Mtr reaction. The presence of that reaction can produce erroneous prediction. Thus, we turned off the MTR_BYPASS reaction in all the simulations by adding the following constraints:

(4) $$v_{MTR\_BYPASS}=0\;\;\;\;\;\;\;\;\;\;\;\;\;\;\;\;\;\;\;\;\;\;$$

Additionally, *rnf* is an essential enzyme when acetate is the substrate [1]. However, for methanol, rnf is not an essential reaction [1]. To model these two conditions, we have added the following constraint to the model:

(5) $$v_{rnf}=f\cdot v_{fpo}\;\;\;\;\;\;\;\;\;\;\;\;\;\;\;\;\;\;\;\;$$

Here, f is the fraction that relates flux trhough rnf and fpo. To simulate ΔhdrABC mutant, we incorporated the following constraint in the model.

(6) $$v_{hdrABC}\;\;=\;\;\;\;\;0\;\;\;\;\;\;\;\;\;\;\;\;\;\;\;\;\;\;\;\;\;\;\;\;\;\;\;\;\;$$

For growth on acetate uptake only, we set the acetate uptake rate as $10\frac{mmol}{gDW.hr}$. Similarly, for the growth on methanol uptake only, we set the methanol uptake rate as $10\frac{mmol}{gDW.hr}$.

The General Algebraic Modeling System (GAMS) version 24.7.4 with IBM CPLEX solver was used to run pFBA algorithm in a Linux-based high-performance cluster computing system at the University of Nebraska-Lincoln.

## 3. Results

### 3.1. Sulfide Partially Rescues the ΔhdrABC Mutant Phenotype on Methanol

**Figure 2:**
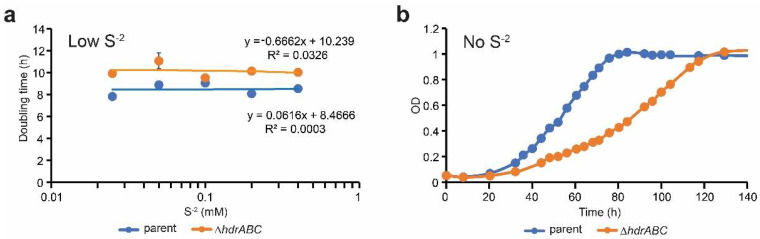
Effect of sulfur source on growth of the ΔhdrABC mutant on methanol. (**a**) doubling times in hours for the parent (blue) and Δ*hdrABC* mutant (orange) strains in low sulfide culture medium with sodium sulfide concentrations of 0.025 Mm–0.4 mM. Error bars showing standard deviation may be obscured by the symbols. The linear trendline and Pearson R^2^ coefficients are shown indicating no relationship between the doubling time and sulfide concentration for either strain. (**b**) growth of parent (blue) and Δ*hdrABC* mutant (orange) strains over time in culture medium in which sodium sulfide has been omitted. Averages were calculated from a minimum of three independent biological replicates per treatment. Error bars have been omitted for clarity. OD, optical density at 600 nm.

Previous transcriptomic data indicated that deletion of Δ*hdrABC* resulted in increased expression of methanethiol and dimethylsulfide methyltransferases during methylotrophic growth and it was interpreted to be the result of sulfide interacting with Mcr or methyl-corrinoid proteins due to a lack of free CoM-SH for the CoM-SH:methylcorrinoid methyltransferase reaction. It was observed that methanethiol and dimethylsulfide accumulate in cultures and enters the methylotrophic methanogenesis pathway at methyl-H_4_MPT. By this logic, growth of the Δ*hdrABC* mutant strain should be improved by the presence of sulfide in the culture medium. We tested this hypothesis by varying sulfide content in cultures and measuring population growth rates (Table 2). We observed no difference when sulfide concentration was 0.025–0.4 mM, or 0.25x–4x what is routinely used in culture medium (Figure 2a). Across this range of sulfide concentration, the parent cultures had a doubling time of 8.5 ± 0.54 h and the Δ*hdrABC* mutant cultures had a doubling time of 10.1 ± 0.70 h (*p* = 0.001), which is 19% slower. However, when strains were grown using cysteine as sole sulfur source [10], the growth rate defect of the Δ*hdrABC* deletion mutant was severely affected. In comparison to the parental strain, which had a population doubling time of 15.3 ± 1.95 h, the Δ*hdrABC* mutant had a doubling time of 27.8 ± 3.62 h, an 81% increase in population doubling time. These results suggest that 0.025 mM sulfide is sufficient for optimal growth of *M. acetivorans* and that levels up to 0.4 mM sulfide are not beneficial or detrimental to cells. The positive growth effect of sulfide is beneficial to the Δ*hdrABC* strain, but the effect is not enhanced above 0.025 mM sulfide, and sulfide alone cannot completely rescue the mutant phenotype.

### 3.2. Mixotrophic Growth on Methanol + Acetate Does Not Rescue the DhdrABC Mutant Phenotype

Next, we tested whether mixotrophic growth could rescue the Δ*hdrABC* mutant phenotype. The Δ*hdrABC* mutant strain is a knockout deletion of the methylotrophic-specific *hdrA1B1C1* locus but it still has the ferredoxin/F_420_H_2_:CoM-S-S-CoB HdrABC heterodisulfide reductase genes encoded by the *hdrA2*/polyferredoxin and *hdrC2B2* loci. We hypothesized that acetate supplementation may induce expression of *hdrA2:polyferredoxin* and *hdrC2B2* genes, and thus compensate for the lack of the *hdrA1B1C1* locus in the Δ*hdrABC* mutant. Additionally, HdrA1B1C1 is proposed to directly or indirectly cooperate with Rnf during methylotrophic growth to oxidize ferredoxin produced by Cdh when catalyzing the oxidation of methylene-H_4_MPT to CHO-MF. Therefore, it was hoped that acetate supplementation would result in increased *rnf* expression that could also compensate for the lack of HdrA1B1C1.

**Figure 3:**
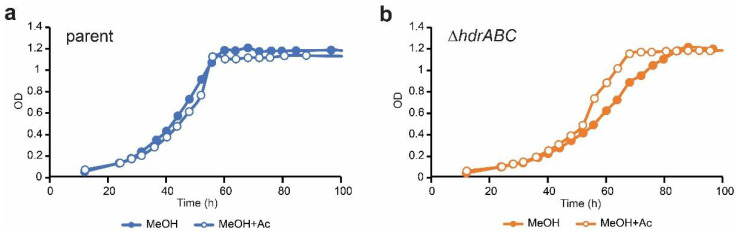
Effect of acetate supplementation on growth of the Δ*hdrABC* mutant. (**a**) growth curves for the parent (blue) on methanol as energy source (closed circles) and methanol with acetate as energy sources (open circles). (**b**) growth curves for the Δ*hdrABC* mutant (orange) on methanol as energy source (closed circles) and methanol with acetate as energy sources (open circles). Averages were calculated from a minimum of three independent biological replicates per treatment. Error bars have been omitted for clarity. OD, optical density at 600 nm.

However, acetate supplementation did not significantly affect growth of the Δ*hdrABC* mutant. The growth rates of both the parent and the Δ*hdrABC* mutant strains were the same in methanol versus methanol + acetate cultures (Table 3). Although there was a small improvement in growth rate with acetate supplementation, the difference was not statistically significant (12.6 ± 1.01 vs. 11.8 ± 0.26, *p* = 0.18 for Δ*hdrABC* mutant on MeOH vs. MeOH+acetate, respectively) and the difference between the parent and the Δ*hdrABC* mutant strain on MeOH+acetate was statistically different (10.8 ± 0.14 vs. 11.8 ± 0.26, *p* = 0.00 for the parent vs. Δ*hdrABC* mutant on MeOH+acetate, respectively). We did observe a difference in the shape of the curve between methanol only and methanol + acetate growth curves for the Δ*hdrABC* mutant; however, growth rates were statistically indistinguishable from growth on methanol alone, as was the final optical density (Figure 3). We interpret these results to suggest that mixotrophic growth on methanol + acetate alone does not completely compensate for the lack of Δ*hdrABC*.

### 3.3. CoM Supplementation Alone Does Not Improve Methylotrophic Growth

**Figure 4:**
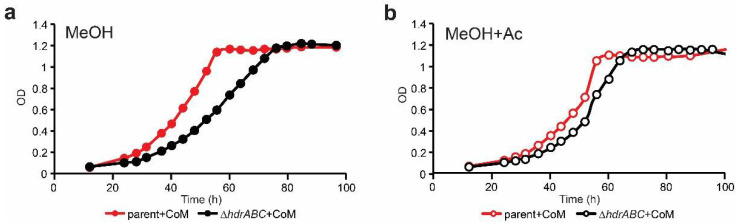
Effect of CoM-SH and acetate supplementation on growth of the ΔhdrABC mutant. (**a**) growth of parent (red) and Δ*hdrABC* mutant (black) strains on methanol as energy source (closed circles) with 1 mM CoM-SH supplementation. (**b**) growth of parent (red) and Δ*hdrABC* mutant (black) strains on methanol plus acetate (open circles) with 1 mM CoM-SH supplementation. Averages were calculated from a minimum of three independent biological replicates per treatment. Error bars have been omitted for clarity. OD, optical density at 600 nm.

A competing explanation for the reduced growth rate of the Δ*hdrABC* mutant strain is that deletion of the methylotrophic CoM-S-S-CoB heterodisulfide results in accumulation of CoM-S-S-CoB heterodisulfide and a decrease in free CoM-SH, thus causing a kinetic bottleneck in methanol:CoM methyltransferase activity. In this scenario, an intracellular increase in CoM-SH could rescue the growth defect. To test this hypothesis, we supplemented methanol cultures with 1 mM CoM-SH. The addition of CoM-SH had no effect on the growth rate of either the parent strain or the Δ*hdrABC* mutant (Figure 4a). Although CoM-SH supplementation decreased the average doubling time for the Δ*hdrABC* mutant from 12.6 ± 1.01 h to 11.5 ± 0.22 h, the difference was not significant (*p* = 0.2) due to the high observed variability in cultures grown without CoM-SH supplementation (Table 3).

### 3.4. CoM-SH and Acetate Supplementation Phenotypically Compensate for Lack of HdrABC

When methanol cultures were provided with acetate and CoM-SH together the growth phenotype of the Δ*hdrABC* mutant was significantly improved and was statistically indistinguishable from the parent strain (Figure 4b). The growth rate of the parent strain was 10.3 ± 0.21 h versus 10.1 ± 0.29 h for the Δ*hdrABC* mutant (*p* = 0.68). These data were interpreted to suggest that the Δ*hdrABC* mutant may have slower growth on methanol because free CoM-SH is depleted, which results in slower rates of carbon fixation that can be reversed by the addition of acetate.

### 3.5. In Silico Analysis for Metabolic Bypass of HdrABC Activity by Acetate and Sulfide

**Figure 5:**
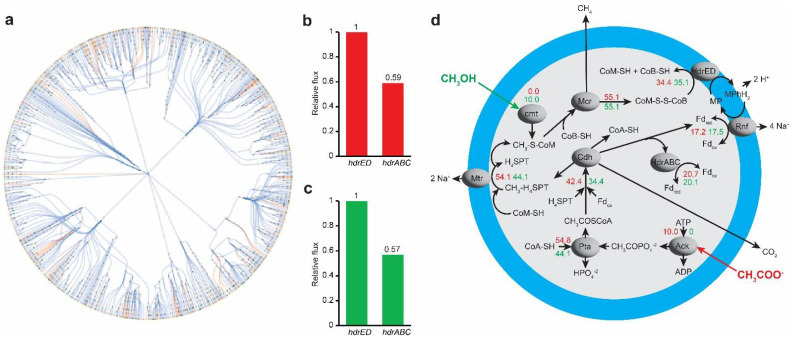
In silico analysis of the electron transport chain of M. acetivorans. (**a**) represents the metabolic network of *iST807* which was generated using fluxer [11]. (**b**) represents the relative reaction flux of *hdrABC* compared to *hdrED* when acetate is the carbon source. (**c**) represents the relative reaction flux of *hdrABC* compared to *hdrED* when methanol is the carbon source. (**d**) represents the reaction flux distribution for $10\frac{mmol}{gDW.hr}$ of acetate uptake (red numbers) and for $10\frac{mmol}{gDW.hr}$ of methanol uptake (green numbers).

To analyze the metabolic trade-offs of the Δ*hdrABC* mutant, we used the most recent genome-scale metabolic model (GEM) of *M. acetivorans*, *iST807* [8]. The metabolic network of *iST807* can be visualized in Figure 5a. The *iST807* model includes both classes of Hdr, cytoplasmic HdrABC and membrane-bound HdrED, but does not distinguish between the two versions of HdrABC expressed from the *M.acetivorans* chromosome: a methylotrophic-specific HdrA1B1C1 (studied here) and an essential constitutive electron-bifurcating HdrA2C2B2. Using the *iST807* model, growth on acetate uptake and methanol uptake were analyzed for wild type and Δ*hdrABC* mutant of *M. acetivorans* in the absence vs. presence of dimethyl sulfide.

For growth on acetate the model did not initially predic flux through *ack* and *pta*. Thus, there must be some other source(s) of acetoacetyl-CoA, which is required to produce 5-Methyl-H_4_SPT. Further investigation of the model revealed that acetoacetyl-CoA produced from the central metabolic pathway (*por*) and cysteine metabolism (*cysE*) contributed to the all the acetoacetyl-CoA requirements. Once *por* and *cysE* were turned off, acetoacetyl-CoA was produced through *ackA* and *pta*.

For acetate uptake, *iST807* predicted that the acetate-specific *hdrABC* and *hdrED* carried reaction flux in a ratio of 0.59:1 (Figure 5b). When *hdrABC* was deleted, all the required oxidized ferredoxin was produced by *hdrED* alone and biomass growth was unchanged. However, from experimental results [1], it is known that the *hdrA1B1C1* genes are not expressed while *hdrA2B2C2* and *hdrED* genes are essential during acetotrophic growth. Thus, to match experimental prediction, we deleted the activity of *hdrABC*. Once we deleted the activity of *hdrABC*, *hdrED* became essential, and reduced flux through hdrED resulted in reduced biomass growth rate.

For growth on methanol, *hdrABC* and *hdrED* carried reaction flux in a ratio of 0.57:1, respectively (Figure 5c). However, while growing on methanol, if *pta*, *cdh*, and formate formation from carbon monoxide reactions are turned off, all the oxidized ferredoxin is produced by *hdrABC* (by the combined action of HdrA1B1C1 and HdrA2B2C2 enzymes), and *hdrED* does not carry any flux. However, the activity of *hdrED* is essentail to *M. acetivorans.* Thus, we stopped the reaction fluxes through *hdrABC*, and only allowed *hdrED* to carry flux. When dimethyl sulfide is added to the model, growth rate on methanol or acetate does not increase unless *hdrABC* is allowed to carry flux. In practice, higher flux through *hdrABC* could be achieved by increased expression or substrate fluxes (reduced methanophenazine and/or CoM-S-S-CoB) and may vary depending on environmental conditions. 

Mixotrophic growth The flux distribution of wild type and Δ*hdrABC* mutant for $10\frac{mmol}{gDW.hr}$ of acetate uptake and $10\frac{mmol}{gDW.hr}$ of methanol uptake is shown in Figure 5d without knocking out *hdrABC*. These data suggest during mixotrophic growth on methanol + acetate, fluxes through *ack, pta, cdh, mtr* and *hdrABC* increase due to the presence of acetate while fluxes through corrinoid methyltransferases*, rnf,* and *hdrED* increase due to methanol (or sulfide) resulting in overall increased flux through energy-conserving reactions of the methanogenesis pathway.

## 4. Discussion

**Figure 6:**
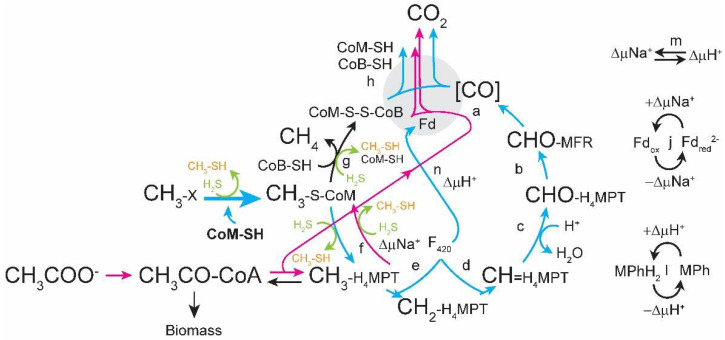
Model for complementation of ΔhdrA1B1C1 function by supplementation with CoM-SH, acetate, and sulfide. Abbreviations and enzymes are as indicated in Figure 1. Exogenously supplied CoM-SH is indicated in bold. Pink arrows indicate acetotrophic methanogenesis pathway. Note several steps are bi-directional between methylotrophic and acetotrophic pathways. Figure adapted from [3].

Our findings show the surprising result that decreased HdrABC enzyme activity can be phenotypically overcome by addition of sulfide or a combination of supplementation with CoM-SH and acetate during methylotrophic growth. These results suggest two mechanisms, which are not mutually exclusive (Figure 6): (1) increased sulfide may result in conversion of methanol to methylsulfides and increased expression of corrinoid methyltransferases that better captures substrate for conversion to methyl-CoM, and (2) a combination of faster conversion of methanol to methyl-CoM by adding exogenous CoM-SH with expression of acetate-specific genes either results in increased flux through HdrA2B2C2 to compensate for Δ*hdrABC* and/or an increase in metabolic efficiency. Metabolism of acetate requires acetate kinase and phosphotransacetylase enzymes with hydrolysis of ATP to ADP. In contrast, during growth on methanol, acetyl-CoA is generated by fixing CO_2_ and coenzyme A (CoA-SH) with methyl-H_4_MPT and uses two electrons. Thus, using acetate to produce acetyl-CoA during mixotrophic growth on methanol + acetate requires more energy than when growing on methanol alone. The energy demand could be compensated for by increased flux through the electron-bifurcating HdrA2B2C2, and/or by efficient sodium pumping by Rnf, both of which are up-regulated in the presence of acetate [8,12]. Future experiments with combinations of mutations and culture conditions are needed to test these hypotheses.

*M. acetivorans* is an attractive methanogenic organism to study because it can use multiple methanogenesis pathways (methylotrophic, acetotrophic, and carboxydotrophic). It also has the largest methanoarchaeal genome sequenced to date [13], and a large contingent of unknown or general-function genes predicted that suggests a wealth of undiscovered molecular biology and biochemistry. Although growth experiments in defined medium with limited carbon and energy sources is necessary to develop an understanding of gene:function relationships through genetic and molecular approaches, it is also important to consider higher-order interactions between genes controlling metabolism [14,15,16,17]. Much work has been done to uncover the differences in transcript abundance between growth using single energy sources in *Methanosarcina* genera [8,18,19,20,21,22,23], but is unknown how the need to transition from one growth substrate to another is sensed, and how changes in culture medium composition affect metabolic fluxes. Our study highlights the need for further experiments to understand how genetics and environmental factors such as sulfur source and mixotrophic growth interact to affect growth of methanogens to better understand how methanogens compete with each other and with other microbes for nutrients in natural microbial communities [14,24,25,26].

## 5. Summary and Conclusions

Physiology and computational modeling experiments presented in this work demonstrate that environmental conditions affect metabolic flux through methanogenesis pathway enzymes. In this study, we show supplementation of sulfide or a combination of acetate and CoM-SH can overcome the growth rate defect caused by deletion of the methylotrophic HdrABC genes. Computational modeling supports the interpretation that relative fluxes through Hdr enzymes (HdrA1B1C1, HdrA2B2C2 and HdrED) change depending on composition of the culture medium, and that mixotrophic growth on both methanol plus acetate may increase flux through energy-conserving enzymes Mtr and Rnf as compared to growth on either substrate alone.

## Figures and Tables

**Table 1:** Primers and strains used in this study.

Primers			
*Name*	*Sequence*	*Purpose*	*Source*
oNB121	GCACCCAGGCACATTGTTC	*hdrA* 301 rev	[2]
oNB122	TACTGGGGTTTCTGGGAGAC	*hdrA* 1024 rev	[2]
oNB123	ATGCCCTCTCCGTAAATGAG	*hdrA* 1880 fwd	[2]
oNB124	GATTCAAGCACACTGCGATC	*hdrC* 2616 rev	[2]
*Methanosarcina acetivorans* C2A strains			
34	*parent*	Δ*hpt::φC31 int attP*	[7]
36	Δ*hdrABC*	Δ*hpt::φC31 int attP ΔhdrA1B1C1*	[1]

**Table 2:** Effect of sulfide concentration on growth rate.

Strain	S^−2^ (mM)	Doubling Time (h) ^a^	Std Dev ^a^	P vs. Parent ^b^	P vs. *DhdrABC* ^b^
parent	0 ^c^	15.3	1.95	0.003	0.014
	0.025	7.8	0.27	0.003	0.001
	0.05	8.9	0.29	0.478	0.026
	0.1^d^	9.1	0.33	1	0.126
	0.2	8.1	0.32	0.010	0.002
	0.4	8.5	0.21	0.038	0.001
*DhdrABC*	0 ^c^	27.8	3.62	0.001	0.006
	0.025	9.9	0.24	0.023	0.152
	0.05	11.1	0.73	0.005	0.031
	0.1 ^d^	9.5	0.21	0.126	1
	0.2	10.1	0.20	0.009	0.042
	0.4	10.0	0.09	0.343	0.034

^a^ Averages were calculated from a minimum of three independent biological replicates per treatment. ^b^ *p* value > 0.05 is not deemed statistically significant. ^c^ Cultures were adapted for 15 generations into culture medium without resazurin or sodium sulfide. ^d^ Routine culture medium contains 0.1 mM sodium sulfide.

**Table 3:** Effect of acetate and coenzyme M supplementation on growth rates.

Strain	Energy Source ^a^	CoM-SH ^b^	Doubling Time (h) ^c^	Std Dev ^c^	P vs. Parent ^d,e^	P vs. Δ*hdrABC* ^e,f^
parent	MeOH	−	9.2	0.35	1	
		+	10.1	0.15	0.01	
	MeOH+Ac	−	10.8	0.14	0.00	
		+	10.3	0.21	0.00	
*DhdrABC*	MeOH	−	12.6	1.01	0.00	1
		+	11.5	0.22	0.00	0.20
	MeOH+Ac	−	11.8	0.26	0.00	0.18
		+	10.1	0.29	0.68	0.00

^a^ MeOH, 125 mM methanol. MeOH + Ac, 125 mM methanol + 40 mM acetate. ^b^ −, no supplementation; +, supplemented with 1 mM coenzyme M. ^c^ Averages were calculated from a minimum of three independent biological replicates per treatment. ^d^ Compared to parent strain grown on 125 mM methanol. ^e^ *p* value > 0.05 is not deemed statistically significant. ^f^ Δ*hdrABC* mutant strain is compared to itself when grown on 125 mM methanol.

## Data Availability

All growth data that support the findings of this study are provided in the manuscript.
